# P57 What are the outcomes of persistent oligoarthritis? A systematic review plan

**DOI:** 10.1093/rap/rkac067.057

**Published:** 2022-09-28

**Authors:** Andrew Smith, Caledonia Swindells-Macleod, Lianne Kearsley-Fleet, Kimme Hyrich, Stephanie Shoop-Worrall

**Affiliations:** Centre for Epidemiology Versus Arthritis, Centre for Musculoskeletal Research, The University of Manchester, Manchester, United Kingdom; NIHR Manchester Biomedical Research Centre, Manchester University NHS Foundation Trust, Manchester Academic Health Science Centre, Manchester, United Kingdom; Centre for Epidemiology Versus Arthritis, Centre for Musculoskeletal Research, The University of Manchester, Manchester, United Kingdom; NIHR Manchester Biomedical Research Centre, Manchester University NHS Foundation Trust, Manchester Academic Health Science Centre, Manchester, United Kingdom; Centre for Epidemiology Versus Arthritis, Centre for Musculoskeletal Research, The University of Manchester, Manchester, United Kingdom; Centre for Epidemiology Versus Arthritis, Centre for Musculoskeletal Research, The University of Manchester, Manchester, United Kingdom; NIHR Manchester Biomedical Research Centre, Manchester University NHS Foundation Trust, Manchester Academic Health Science Centre, Manchester, United Kingdom; Centre for Epidemiology Versus Arthritis, Centre for Musculoskeletal Research, The University of Manchester, Manchester, United Kingdom; Centre for Health Informatics, The University of Manchester, Manchester, United Kingdom

## Abstract

**Introduction/Background:**

Oligoarthritis, a sub-type of JIA, can be categorised as persistent or extended. Children with persistent oligoarthritis, involving <5 joints, are often excluded from clinical trials as it is considered a milder form of JIA and managed less aggressively. Therefore, less is known about the longer term outcomes of those with persistent oligoarthritis. This knowledge could lead to better targeting of existing therapies for individuals and higher quality patient information.

**Description/Method:**

This aim of this research is to conduct a systematic literature review on the clinical and patient-reported outcomes of persistent oligoarthritis. This abstract presents plans and current status of the review.

Inclusion criteria for this systematic review are 1) inception cohorts with at least partial prospective data collection, 2) available in English language, 3) includes at least 50 patients with persistent oligoarthritis using ILAR criteria, 4) investigates clinical or patient-reported outcome. Exclusion criteria are 1) not specifically presenting or analysing outcomes in persistent oligoarthritis and 2) not reporting disease duration or follow-up time points from diagnosis at the point of outcome measurements.

Search terms related to ‘oligoarthritis’, (variants of juvenile oligoarthritis plus outcomes of interest) were used in MEDLINE and Embase from 01/01/1997 to 30/05/2022 to cover the advent of current ILAR categories to present. These terms were also entered into PubMed from 30/05/2020 to 30/05/2022 to include publications published online ahead of print.

Quality assessment, including common biases, of selected articles will be assessed using relevant questions from the Pasma et al. quality assessment (QA) tool.

Primary outcomes are the core JIA outcome variables (active joint counts, limited joint count, physician’s global assessment, parent global evaluation, erythrocyte sedimentation rate and additional functional status (CHAQ/HAQ). Additional outcomes are 1) remission status, 2) uveitis status, 3) pain, 4) Health-related Quality of Life and 5) treatment response.

One reviewer will initially screen titles and abstracts. Two reviewers will then screen the full text of selected articles independently and agree the master list. The first reviewer will then extract data regarding study populations and outcome and another reviewer will check a selection of the extracted data. If there is disagreement or uncertainty between the two reviewers at any stage, a third reviewer will adjudicate.

**Discussion/Results:**

To date 1051 publications have been highlighted for initial review (title and abstract) by the first reviewer. The second review is now underway by the two reviewers and preliminary results will be ready to present soon. The review has been registered with Prospero (ID: CRD42022291943).

**Key learning points/Conclusion:**

There is an unmet need for improved understanding of outcome in persistent oligoarthritis. This systematic review will help inform treatment targets and future research in this common ILAR JIA category.

